# Functional magnetic resonance spectroscopy of glutamate in schizophrenia and major depressive disorder: anterior cingulate activity during a color-word Stroop task

**DOI:** 10.1038/npjschz.2015.28

**Published:** 2015-09-16

**Authors:** Reggie Taylor, Richard W J Neufeld, Betsy Schaefer, Maria Densmore, Nagalingam Rajakumar, Elizabeth A Osuch, Peter C Williamson, Jean Théberge

**Affiliations:** 1 Department of Medical Biophysics, University of Western Ontario, London, ON, Canada; 2 Lawson Health Research Institute, London, ON, Canada; 3 Department of Psychiatry, University of Western Ontario, London, ON, Canada; 4 Department of Psychology, University of Western Ontario, London, ON, Canada; 5 Department of Neuroscience, University of Western Ontario, London, ON, Canada; 6 Department of Anatomy and Cell Biology, University of Western Ontario, London, ON, Canada

## Abstract

**Background:**

Glutamate abnormalities have been suggested to be associated with symptoms of schizophrenia. Using functional magnetic resonance spectroscopy (^1^H-fMRS), it is possible to monitor glutamate dynamically in the activated brain areas, which has yet to be reported in schizophrenia. It was hypothesized that subjects with schizophrenia would have weaker glutamatergic responses in the anterior cingulate to a color-word Stroop Task.

**AIMS:**

The aim of this study was to gain insight into the health of GLU neurotransmission and the GLU-GLN cycle in SZ using a ^1^H-fMRS protocol.

**Methods:**

Spectra were acquired from the anterior cingulate of 16 participants with schizophrenia, 16 healthy controls and 16 participants with major depressive disorder (MDD) while performing the Stroop task in a 7T magnetic resonance imaging scanner. ^1^H-fMRS spectra were acquired for 20 min in which there were three 4-min blocks of cross fixation interleaved with two 4-min blocks of the Stroop paradigm.

**Results:**

A repeated-measures analysis of variance revealed a main effect of time for glutamate concentrations of all groups (*P*<0.001). The healthy control group increased glutamate concentrations in the first run of the Stroop task (*P*=0.006) followed by a decrease in the recovery period (*P*=0.007). Neither the schizophrenia (*P*=0.107) nor MDD (*P*=0.081) groups had significant glutamate changes in the first run of the task, while the schizophrenia group had a significant increase in glutamine (*P*=0.005). The MDD group decreased glutamate concentrations in the second run of the task (*P*=0.003), as did all the groups combined (*P*=0.003).

**Conclusions:**

^1^H-fMRS data were successfully acquired from psychiatric subjects with schizophrenia and mood disorder using a cognitive paradigm for the first time. Future study designs should further elucidate the glutamatergic response to functional activation in schizophrenia.

## Introduction

Schizophrenia (SZ) has been suggested to be associated with dysfunction in brain areas that utilize glutamate (Glu) for neurotransmission.^1^ It has been postulated that Glu *N*-methyl-D-aspartate receptor hypofunction may contribute to symptoms of SZ^2–4^ and this body of literature has been recently reviewed.^5,6^ This, and other lines of evidence, has led to the notion that Glu abnormalities can explain a wider range of symptoms of SZ than dopamine abnormalities alone, thus characterization of Glu abnormalities in SZ is sorely needed in light of potential glutamate-modulating treatment strategies.^6^

Proton magnetic resonance spectroscopy (^1^H-MRS) has demonstrated abnormal brain Glu concentrations, with its metabolic precursor/by-product, glutamine (Gln), in individuals with SZ.^7,8^ However, findings may not be specific to this illness. Both major depressive disorder (MDD) and bipolar disorder have demonstrated abnormalities of Glu in multiple brain areas using ^1^H-MRS.^9–11^ Although MDD has been consistently reported to have lower glutamatergic metabolites (Glu, Gln, or Glx (Glu+Gln)), bipolar disorder has demonstrated inconsistent results, with a tendency to be elevated.^9–11^ The consistency of studies of Glu in MDD makes it a preferable choice for a psychiatric control group.

An increasing number of studies demonstrate the utility of functional ^1^H-MRS (^1^H-fMRS) in dynamic measures of metabolic content.^12–22^ Much like its parent technique, ^1^H-MRS, ^1^H-fMRS assesses concentrations of brain metabolites that are orders of magnitude smaller than the water content. Essentially, ^1^H-fMRS is a time course of ^1^H-MRS spectra that typically measures slow metabolic responses to prolonged stimuli in a small volume of tissue within the brain.^13–16,18–20^

Increases in concentrations of glutamatergic metabolites have been demonstrated in ^1^H-fMRS studies of healthy controls in the occipital lobe using visual stimuli,^14,16,17,19^ the motor cortex using a finger tapping paradigm^18^ and the anterior cingulate cortex (ACC) using pain paradigms,^21,22^ a sexual arousal paradigm,^13^ and the color-word Stroop task.^20^ Increases in glutamatergic metabolites upon stimulation is not surprising given the tight coupling of Glu and Gln cycling to neural response.^23^ ^1^H-fMRS with controlled rest and activation periods could provide unique information about the dynamic nature of glutamatergic abnormalities in SZ.

In the simplest of tasks, stimuli received by the brain must be organized and encoded for further use by brain centers involved in cognition. It is postulated that deficits of stimulus encoding are central to cognitive deficits in schizophrenia.^24,25^ Previous literature using the Stroop task in healthy controls and in SZ has shown that this is a task that both participant groups conduct to the same level of proficiency (rate of correct answers) and robustly activates the ACC, although with some hypofunction in SZ.^26,27^

The purpose of this study is to measure ACC glutamatergic concentrations dynamically during the performance of a color-word Stroop task in SZ compared with healthy controls and psychiatric controls with MDD using a ^1^H-fMRS technique. It is hypothesized that there will be smaller Glu responses along with slower response times in the SZ group compared with both the healthy controls and MDD group due to the increased number of encoding subprocesses (constituent cognitive operations), indicating involvement of more brain areas as well as diversion of activity away from the ACC.^24,28^ Within groups, it is hypothesized that there will be an increase in Glu concentrations during activation of the ACC, with a subsequent return to baseline after the task. Because neuronal Glu levels have been shown to be related to cognition,^29^ it is expected that concentrations of the glutamatergic metabolites will negatively correlate with the response times.

## Materials and Methods

### Participants

There were 16 participants in each of the healthy, MDD, and SZ groups who gave informed written consent according to the guidelines of the Review Board for Health Sciences Research Involving Human Subjects at the University of Western Ontario. The number of participants was chosen on the basis of previous ^1^H-fMRS studies that have observed glutamate changes of 2–4%.^14,16–20^ Volunteers with neurological or major medical illnesses, clinically significant head injury, other psychiatric disorders, magnetic resonance imaging (MRI) contraindications, or substance abuse within the previous year were excluded from the study. Any healthy volunteer with a known family history of psychiatric disorder in a first- or second-degree relative was also excluded.

A consensus diagnosis was established on all the participants by a psychiatrist and trained assistant with the Structured Clinical Interview for DSM-IV.^30^ SZ subjects were rated with the Scale for Assessment of Negative Symptoms and the Scale for the Assessment of Positive Symptoms^31,32^ and MDD patients were assessed with the Montgomery–Asberg Depression Scale^33^ and the Young Mania Rating Scale.^34^ Fourteen SZ patients were receiving atypical neuroleptics with chlorpromazine equivalent 409±293 mg (three taking olanzapine; quetiapine/venlafaxine; two taking risperidone; quetiapine/paliperidone/escitalopram; four taking paliperidone; clozapine; risperidone/escitalopram; quetiapine/escitalopram) and two patients were not medicated. Ten of the 16 MDD patients were receiving antidepressant medications at the time of the scan (bupropion/citalopram/methylphenidate; venlafaxine; lamotrigine; desvenlafaxine; bupropion/citalopram; escitalopram; citalopram; sertraline; citalopram/mirtazapine/quetiapine; levothyroxine/melatonin). Demographic information including age, handedness, education, parental education, clinical rating scores, and length of illness were collected according to our previous study^35^ and are shown in Table 1.

### Anterior cingulate activation paradigm

We have previously described details of the color-word Stroop Task chosen for the functional paradigm.^20^ In brief, it was a four-condition (congruent, incongruent, word-only, color-only) by four-color (red, green, blue, yellow) design. The subjects were asked to respond as quickly and accurately as possible on a four-button keypad with the color of the ink as the correct answer for all but the word-only conditions within which the answer was the color-word. Stimuli were presented for 2 s followed by 1 s of cross fixation. In the scanner, subjects first engage in cross fixation for 4 min before a 4-min block of activation (Stroop1), which is then followed by 4 min of recovery (Recovery1). In contrast to the previous study by Taylor *et al.,* ^20^ an additional 4 min of Stroop activation (Stroop2) was acquired after Recovery1, which was then followed by another 4-min recovery period (Recovery2). This additional block of activation will help assess the glutamatergic response to repeated, prolonged Stroop stimuli in the ACC. The procedure was written and presented using PsychoPy,^36^ which also recorded the accuracy and response times. A confirmatory fMRI was acquired post-^1^H-fMRS to ensure activation within the fMRS voxel. The fMRI lasted 9 min and was divided into 1-min blocks cycling between resting and Stroop activation, for a total of 4 min of Stroop activation. Image preprocessing and statistical analysis were conducted using Statistical Parametric Mappping (SPM8; Wellcome Department of Neurology, London, UK) within Matlab 7.1 (Mathworks, Natick, MA, USA).

### ^1^H-fMRS data collection and analysis

All the measurements were acquired on a 7.0T Agilent/Magnex head-only MRI (Agilent, Walnut Creek, CA, USA) with a Siemens AC84 head gradient coil (Siemens, Erlangen, Germany), located at the Center for Functional and Metabolic Mapping at Western’s Robarts Research Institute. A transmit-only receive-only head coil with 15 transmitters and 23 receivers^37^ was used for all the scans with a B1-shimming approach to facilitate optimized homogeneity correction of the transmit field for each scan.^38^ The magnetic field uniformity (B0-shim) was adjusted automatically over the field of view with first-order and second-order shims using RASTAMAP.^39^

**Figure 1:**
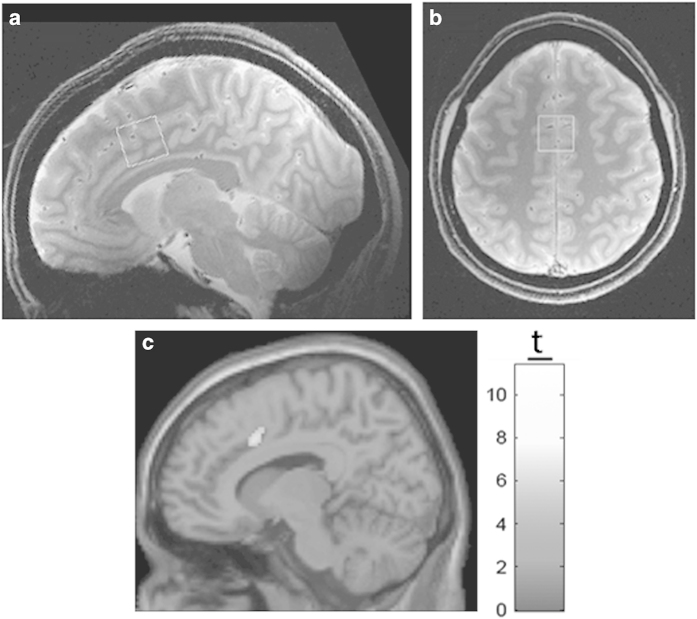
(**a**) Sagittal and (**b**) transverse cross-sections depicting the position of the ^1^H-fMRS voxel located in the bilateral ACC in the native space of one participant. The voxel was centered on the junction of the right cingulate sulcus with the paracentral sulcus, angled to the AP line and placed superior to the first fold of gray matter above the corpus callosum in the sagittal cross-sections and centered on the interhemispheric fissure in the transverse cross-sections. (**c**) The family-wise error (FWE) corrected (*P*<0.05) confirmatory fMRI on a normalized average brain for all the groups showing significant activation within the ACC in the right hemisphere of all the participant groups and Stroop conditions combined. ACC, anterior cingulate cortex; AP, antero-posterior; fMRI, functional magnetic resonance imaging; fMRS, functional magnetic resonance spectroscopy.

The ^1^H-MRS voxels were 2.0×2.0×2.0 cm (8 cm^3^) in size. In every individual, a voxel was centered medially and encompassed the bilateral ACC (Figure 1) using two fast low-angle shot two-dimensional anatomical imaging sequences in the sagittal (45 slices, repetition time=950 ms, echo time=5.23 ms, flip-angle (*α*)=30°, gap between slices=1 mm, thickness=2 mm, field of view=220×220 mm, matrix size=220×200) and axial (20 slices, repetition time=500 ms, echo time=5.23 ms, flip-angle (*α*)=30°, gap=1 mm, thickness=2 mm, field of view=220×220 mm, matrix size=220×220) directions, both with lipid saturation. The voxels were placed in the areas of the ACC where activation was expected based on previous fMRI studies that used a color-word Stroop task.^40,41^

^1^H-MRS spectra were acquired individually throughout the Stroop paradigm using an ultra-short echo time stimulated echo acquisition mode sequence with outer volume suppression^42^ (repetition time=3 s, echo time=10 ms, mixing time=32 ms, 4,000 complex pairs, four steady state scans, 1 s acquisition time, eight-step phase cycle) with 16 water-unsuppressed spectra and 400 water-suppressed spectra, 80 spectra for each 4-min section of the Stroop paradigm (Resting, Stroop1, Recovery1, Stroop2, Recovery2). An eight-pulse VAPOR preparation sequence, with an additional water suppression pulse during the mixing time period,^42^ provided efficient water suppression. A separate metabolite-suppressed spectrum was acquired to assess the macromolecular content^43^ for each individual. This metabolite-suppressed spectrum was modeled using a Hankel–Lanczos singular value decomposition^44,45^ routine and included in the fitting template. Each acquisition produced 23 spectra, one for each receiver, which required channel combination before use.^46^ Spectra were frequency and phase corrected before being averaged together. Quality Eddy Current Correction (QUECC)^47^ reduced linewidth distortions before spectral fitting with fitMAN, a time-domain fitting algorithm.^48^ Metabolite concentrations were calculated with corrections for gray and white matter content, as previously described in Stanley *et al.* ^49^ All spectra were inspected visually for quality. Only metabolites with Cramer–Rao lower bounds <10% were included in the analysis.

To illustrate the dynamic response of Glu throughout the acquisition, the spectra were subdivided and averaged into 20 spectra (1-min intervals). These were fit for each person, then combined via a moving average for each group.

A 5×3 repeated-measures analysis of variance design using the metabolite concentrations at each 4-min (80 spectral averages) section of the functional paradigm was examined using SPSS v.20 (IBM Corp, Armonk, NY, USA) to determine significant variations over time and across groups. One-tailed tests were used for Glu because of the directional hypotheses of concentration increases with activation by the Stroop Task followed by decreases during the recovery. Gln and Glx were similarly explored; Glx, with one-tailed tests as Glu is the main contributor to Glx concentration, and Gln with two-tailed tests. For pairwise comparison, the blocks of the Stroop paradigm were compared with both the previous block and the sequential block. Metabolite changes will similarly be explored with concentrations that are normalized to their resting values. To accommodate multiple comparisons, alpha was divided by four (*P*<0.05/4). Kolmogorov–Smirnov tests were used to ensure the assumption of normality.

## Results

Significant family-wise error corrected (*P*<0.05) activation of the ACC was observed in the confirmatory fMRI (Figure 1c). The activation observed was within the location of voxel placement in the ACC.

**Figure 2:**
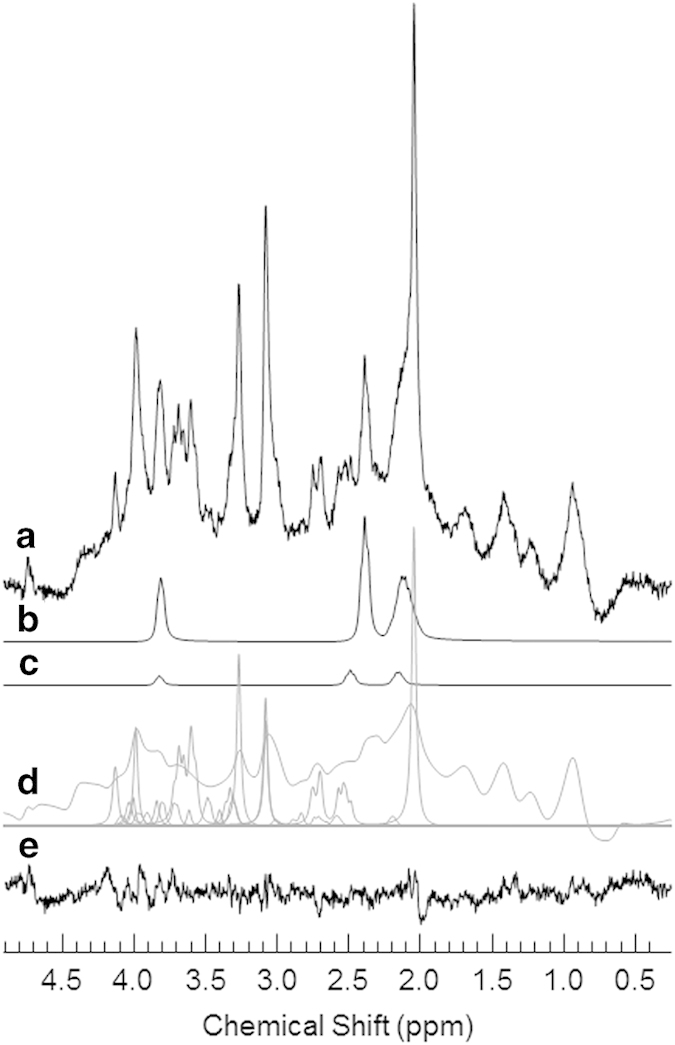
(**a**) An example resting 80 average water-suppressed spectrum from the ACC of a volunteer with SZ. A 1 Hz Lorentzian line broadening has been applied. (**b**) Resultant spectral fits of glutamate, (**c**) glutamine, and (**d**) the remaining metabolites (glutathione, taurine, aspartate, gamma-aminobutyric acid, *N*-acetylaspartylglutamate, *myo*-inositol, *scyllo*-inositol, *N*-acetylaspartate, choline (phosphorylcholine, glycerophosphorylcholine), creatine (phosphocreatine, creatine), glycine, ascorbate) and the macromolecular baseline. (**e**) The residual (data minus the fit of all the metabolites and macromolecules) of the spectrum. ACC, anterior cingulate cortex; SZ, schizophrenia.

**Figure 3:**
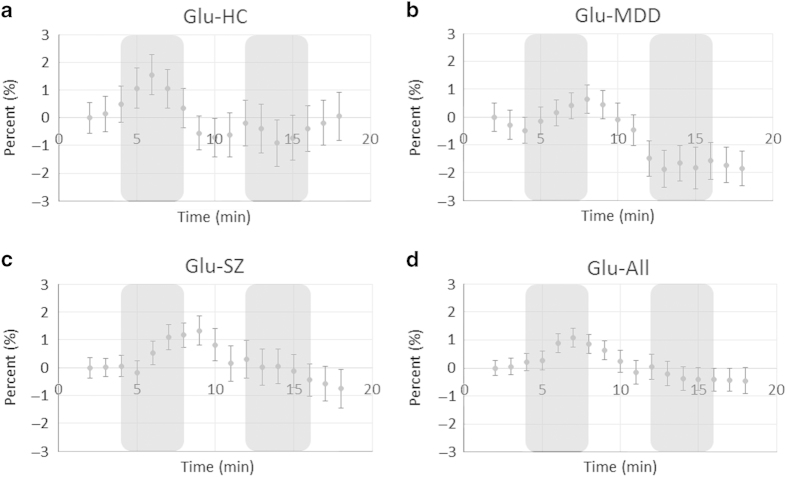
Four-minute moving average time courses of glutamate concentration estimates. Each point represents the percent change from resting concentration (averaged over 4 min) for (**a**) healthy, (**b**) major depressive disorder, (**c**) schizophrenia groups, and (**d**) all the groups combined. Shaded areas indicate that the Stroop Task is being performed during that time. Error bars represent inter-individual standard error of the mean. HC, healthy control; MDD, major depressive disorder; SZ, schizophrenia.

Unsuppressed water spectra were acquired with average linewidths of 10.8±1.1 Hz after shimming and the water peak was effectively suppressed in the metabolite spectra (Figure 2). The Glu and Gln concentrations were estimated from the fit (Figure 2) with Cramer–Rao lower bounds <1 and 10%, respectively, indicating high quality fits of the data. A moving average of fluctuations in the Glu levels throughout the activation paradigm is presented in Figure 3 for each participant group.

The repeated-measures analysis of variance (alpha=0.05) yielded a significant main effect of time for Glu (*P*<0.001) and Glx (*P*<0.001) but not Gln (*P*=0.132). Strongly significant increases occurred for Glu (*P*=0.002) and Glx (*P*=0.001) in Stroop1 with all groups combined together. There were no significant time by group interactions (Glu, *P*=0.377; Gln, *P*=0.317; Glx, *P*=0.616) and there were no main effects of group (Glu, *P*=0.797; Gln, *P*=0.137; Glx, *P*=0.700).

The planned pairwise comparisons (alpha=0.05/4) of adjacent periods of the Stroop Task yielded significantly increased Glu (*P*=0.006) concentrations in the healthy controls during Stroop1 (Table 2). Glu then decreased towards the resting value in Recovery1 (*P*=0.007). In the SZ group, Glx concentrations had a trend to increase Stroop1 (*P*=0.016). The SZ group was the only group to show significant Gln changes, going from 1.21±0.52 mmol/kg_ww_ at rest to 1.44±0.50 mmol/kg_ww_ (*P*=0.004, two-tailed) in Stroop1, then returning to 1.18±0.48 mmol/kg_ww_ in Recovery1 (*P*=0.001, two-tailed). The MDD group did not show any significant changes in the glutamatergic concentrations during Stroop1.

Stroop2 yielded unexpected decreases in Glu and Glx concentrations relative to Recovery1. Using two-tailed tests (as the assumptions for one-tailed tests were no longer valid), the MDD group showed significantly decreased Glu and Glx (*P*=0.003, *P*=0.006, respectively) and the SZ group showed a near-significant decrease for Glu (*P*=0.024). Combining all the groups yielded significant decreases in Glu (*P*=0.003) and Glx (*P*=0.008). Statistical comparisons using concentrations normalized to resting values yielded highly similar results (Supplementary Table 1). A *post hoc* test (alpha=0.05) comparing the Glu concentrations during the two Stroop conditions indicated lower concentrations in Stroop2 for the MDD group (*P*=0.001) and the SZ group (*P*=0.026) but not the healthy control group (*P*=0.053). Glu concentrations in Stroop2 were found to be lower in every group when the concentrations were normalized to the resting values (*P*=0.009, *P*=0.002, *P*=0.034 for control, MDD, and SZ groups, respectively).

Every group was able to respond to the stimuli correctly with at least 90% accuracy. Mathematical modeling of Stroop performance confirmed an increased number of subprocesses (constituent cognitive operations) among the SZ group (Taylor R, Théberge J, Williamson PC, Neufeld RWJ, unpublished data; Taylor R, Théberge J, Williamson PC, Densmore M, Neufeld RWJ, unpublished data).^24,25^ The response times to the incongruent condition during Stroop1 significantly correlated with Gln concentrations when averaged together over all the groups (*P*=0.005; Table 3), whereas Glu concentrations only presented with a trend (*P*=0.017). The responses of the MDD and SZ groups significantly correlated with the normalized Gln responses (*P*=0.003, *P*=0.006, respectively), whereas no group's response times were significantly correlated with the normalized Glu responses. No significant correlations were observed in Stroop2. The full table of correlations between Glu and Gln concentrations and response times for each of the four Stroop conditions can be found in Supplementary Table 2.

## Discussion

The Glu response in the ACC of healthy controls (3.2%) compares well with the 2.6% reported previously using the Stroop Task^20^ and with the 2–4% reported previously in the other brain areas.^14,16–19^ A main effect of time was observed for all the groups in Stroop1 compared with Resting, nevertheless, planned contrasts were significant for the healthy controls only. The Glu and Gln cycling correlates with neuronal glucose consumption in activated conditions^23^ and hypofunction of the ACC with color-word Stroop Tasks has been demonstrated in SZ with fMRI.^26,27^ In SZ, it seems that the number of processing steps involved in stimulus encoding is increased (Taylor R, Théberge J, Williamson PC, Neufeld RWJ, unpublished data; Taylor R, Théberge J, Williamson PC, Densmore M, Neufeld RWJ, unpublished data).^24,25^ The increased processing steps may involve more brain areas than in controls and cause a blunted activation state of the ACC, which could explain the relatively smaller increase in Glu.

The observed decreases in Glu and Glx during Stroop2 relative to Stroop1 and Recovery1, particularly in the patient groups, were unexpected. Learning effects on Glu as the task progresses are a possible explanation owing to the increased amount of practice that the participants received by the time of the second task. The observed decrease in response times during Stroop2 relative to Stroop1 does indicate that the task became easier. This study is now the second to report a decrease in a neurotransmitter using a cognitive task with ^1^H-fMRS, as a similar result has been observed in prefrontal cortex GABA concentrations during a working memory task, which initially showed an increase in GABA concentrations followed by three subsequent runs with decreases.^15^ This finding is not observed in the visual or motor tasks,^14,16–19^ so it is not likely that the reduced Glu is due to an inadequate duration of Recovery1. Future fMRS studies using cognitive stimuli should observe a longer recovery time between functional runs to further explore this finding.

The behavioral response times for the incongruent condition during Stroop1 negatively correlated with the Gln concentrations. More Gln readily available could mean quicker responses are possible because less time is required to create Glu for neurotransmission. The normalized changes in Gln during the task were much stronger predictors than Glu, which seemingly had no correlation, indicating that the role of Gln in neurotransmisison may have substantial influence on cognitive capabilities.

No between-group comparisons yielded significant results but there are still some points worth noting for possible future testing. First, the resting Glu levels were slightly higher in the MDD and SZ groups than in the healthy control group. Elevated Glu concentrations can lead to excitotoxicity,^50,51^ therefore, levels are carefully controlled by re-uptake transporters. It is possible that resting Glu in MDD and SZ groups are closer to a Glu ceiling making Glu upregulation more difficult. It should also be noted that Glu and Gln are involved in many other brain functions, including metabolism, which is likely also in a dynamic state.

Second, the SZ group appeared to have a slower Glu response and recovery to Stroop1 when compared with controls and MDD subjects, yet showed significant differences in the Gln concentrations during Stroop1 and Recovery1. When Glu releases from the receptor on the postsynaptic membrane, it is taken up into the adjacent glial cell^51^ and converted to Gln via glutamine synthetase so that it can be passively transferred back into the pre-synaptic neuron^51^ where the Gln is converted back into Glu via the phosphate-activated enzyme glutaminase.^52^ Insufficient glutaminase would slow the conversion of Gln to Glu, resulting in a slower Glu–Gln cycle and a prolonged recovery from a neurotransmission event. However, no significant difference was observed in the expression of phosphate-activated enzyme glutaminase in the ACC in one post-mortem study^53^ and increased expression was found in the thalamus in another.^54^ Another possible explanation comes from studies that have demonstrated that *N*-methyl-D-aspartate hypofunction causes increased glutamine synthetase activity resulting in increased Gln.^55^ Consistent with this observation, another study has shown that increased ketamine administration in healthy controls leads to increased Gln concentrations in the ACC.^56^ It is possible that the increased Gln observed in the SZ group arose from *N*-methyl-D-aspartate hypofunction when the ACC was challenged.

There is evidence to support a possible glutamatergic dysfunction in MDD as well.^9^ Although the exact mechanism is yet to be understood, some lines of evidence suggest that astrocytic dysfunction may contribute to the pathophysiology of MDD.^9,57,58^ Astrocytes are pivotal elements in the Glu–Gln cycle and it is possible that any disruption to the efficiency of their operations in MDD could have contributed to the lack of a significant increase during Stroop1, or to the significant decrease in Stroop2. Previous studies have demonstrated decreased ACC Glu in the MDD,^9–11^ which was not observed in this study. This could be owing to the different placement of voxels within the relatively large ACC or possibly a result of treatment effects. It could also be due to the significant difference in gender in the subject population. In this study, the MDD group had a larger incidence of females than the healthy controls. This is another confound that is difficult to avoid, as women have a higher prevalence of MDD than males at a ratio of 1.64:1 in Canada.^59^ This must be considered when interpreting the results because gender has previously been shown to influence Glu^60^ and Gln^61^ concentrations.

A limitation of the study is the possible influence of the medications, which have been shown to affect glutamatergic concentration levels.^62,63^ Antipsychotic and antidepressant medications are often unavoidable confounds of studies involving SZ and MDD patients. Although the subjects in this study were in relatively early stages of the illness (30±16 months and 29±14 months for the SZ and MDD groups, respectively), medications have been shown to influence the Glu concentration of medicated SZ patients in as early as 4 weeks.^63^ This could have influenced the baseline levels of Glu and, possibly, the Glu responses to functional activation.

Although the confirmatory fMRI suggests correct voxel placement, another limitation of this study is that there was no fMRI guidance before the fMRS acquisition. The confirmatory fMRI was acquired post-fMRS to decrease the impact of task-learning effects to elicit the strongest ACC response. The reduction in response times and Glu changes observed during Stroop2 do suggest that considerable learning effects occur.

Detailed analyses of the confirmatory fMRI and mathematical modeling of the behavioral response times are beyond the scope of this work and will be presented in other venues (Taylor R, Théberge J, Williamson PC, Neufeld RWJ, unpublished data).

## Conclusion

Glu concentrations measured with ^1^H-fMRS were demonstrated to significantly increase in a healthy control group upon functional activation of the ACC using a color-word Stroop task but not in an MDD or SZ group. This is the first study to perform ^1^H-fMRS in the ACC at 7T in a psychiatric population. Use of a psychiatric control group (MDD) demonstrated that the increases in Gln were specific to SZ, but the blunted Glu response during Stroop1 was not. Observed response times of the MDD group were slower than the healthy controls, but were not as slow as the SZ group, indicating a stronger deterioration of function in SZ. Whenever possible, studies should try to include psychiatric control groups to assess the specificity of the results. Future studies should examine other cognitive tasks that activate the ACC, perhaps with varying levels of complexity to get a better understanding of the Glu and Gln response in SZ, with longer response times and varying stimulus-encoding loads to study the Glu recovery process in greater detail.

## Figures and Tables

**Table 1:** Participant demographics

*Group*	*Controls*	*MDD*	*SZ*	*P*
*n*	16	16	16	
Age	23.9±4.7	21.7±3.3	22.7±2.9	0.234
M/F	11/5	6/10	13/3	**0.030**
R/L	14/2	14/2	15/1	0.810
Educ	3.1±0.9	2.6±0.6	2.2±0.8	**0.010**
PEduc	3.1±0.9	3.0±0.6	3.3±0.8	0.659
HAM-A		12.7±10.9		
HAM-D		12.4±9.1		
Mania		5.4±6.8		
Montg		17.4±10.4		
CPZ (mg)			358±307	
SANS			9.7±7.7	
SAPS			7.6±10.4	
Illness Duration (months)		28.6±14.4	29.5±15.7	

Abbreviations: CPZ, chlorpromazine equivalent; Educ, education rating of the participant (1- gr. 10 or lower, 2- completed high school, 3- 1–3 years of college/university, 4- 43 years of college/university); HAM-A, Hamilton Anxiety Scale; HAM-D, Hamilton Depression Scale; M/F, male/female; Mania, mania rating from the Young Mania Rating Scale; Montg, result of the Montgomery Asperg Depression Scale; SANS, Scale for Assessment of Negative Symptoms; PEduc, education rating of the participant’s parent (1- gr.10 or lower, 2-completed high school, 3- 1–3 years of college/university, 4- 43 years of college/university); R/L, right/left; SAPS, Scale for Assessment of Positive Symptoms.

*P –* ANOVA test for significance (alpha=0.05, two-tailed), bolded values indicate significance.

**Table 2:** Pairwise comparisons for adjacent blocks of the 1H-fMRS paradigm for Glu, Gln, and Glx concentrations

*Group*	*Resting*	*Stroop1*	*p(S1-B)*	*Recovery1*	*p(R1-S1)*	*Stroop2*	*p(S2-R1)*	*Recovery2*	*p(R2-S2)*
*HC*									
[Glu]	8.8±0.9	9.0±0.9	**0.006**	8.8±0.8	**0.007**	8.8±1.1	0.970	8.9±1.1	0.391
[Gln]	1.5±0.4	1.5±0.5	0.541	1.5±0.5	0.950	1.4±0.6	0.538	1.5±0.5	0.853
[Glx]	10.1±1.0	10.4±1.0	0.018	10.2±1.1	0.046	10.1±1.2	0.697	10.2±1.3	0.457
									
*MDD*									
[Glu]	9.2±1.4	9.4±1.5	0.081	9.3±1.5	0.116	8.9±1.5	**0.003**	9.0±1.4	0.716
[Gln]	1.6±0.9	1.6±1.0	0.355	1.7±1.0	0.728	1.6±1.1	0.773	1.5±0.9	0.255
[Glx]	10.6±2.2	10.9±2.4	0.052	10.8±2.5	0.184	10.3±2.5	**0.006**	10.4±2.2	0.794
									
*SZ*									
[Glu]	9.1±1.3	9.2±1.2	0.107	9.2±1.2	0.367	8.9±1.5	0.024	8.9±1.4	0.737
[Gln]	1.2±0.5	1.4±0.5	**0.004**	1.2±0.5	**0.001**	1.2±0.4	0.739	1.2±0.4	0.765
[Glx]	10.2±1.8	10.5±1.7	0.016	10.2±1.6	0.020	10.0±1.9	0.119	10.0±1.8	0.957
									
*All*									
[Glu]	9.1±1.2	9.2±1.2	**0.002**	9.1±1.2	**0.011**	8.9±1.4	**0.003**	8.9±1.3	0.601
[Gln]	1.4±0.7	1.5±0.7	**0.012**	1.4±0.7	0.100	1.4±0.8	0.727	1.4±0.7	0.701
[Glx]	10.3±1.7	10.6±1.8	**0.001**	10.4±1.8	**0.005**	10.1±1.9	**0.008**	10.2±1.8	0.577

Abbreviations: All, the combination of all participants across groups; [Gln], The concentration of glutamine in mmol/kg_ww_; [Glu], The concentration of glutamate in mmol/kg_ww_; [Glx], The concentration of Glx (glutamate + glutamine) in mmol/kg_ww_; HC, Healthy controls; MDD, Major Depressive Disorder; *p(S1-B)*, Stroop1 vs resting, (alpha=0.05/4 (Bonferroni corrected); one-tailed (Glu, Glx), and two-tailed (Gln)), bolded values indicate statistical significance; *p(S2-R1)*, Stroop2 vs Recovery1, (alpha=0.05/4 (Bonferroni corrected); two-tailed (Glu, Gln, Glx)), bolded values indicate statistical significance; *p(R1-S1)*, Recovery1 vs Stroop1, (alpha=0.05/4 (Bonferroni corrected); one-tailed (Glu, Glx), and two-tailed (Gln)), bolded values indicate statistical significance.; *p(R2-S2)*, Recovery2 vs Stroop2, (alpha=0.05/4 (Bonferroni corrected); two-tailed (Glu, Gln, Glx)), bolded values indicate statistical significance; SZ, Schizophrenia

**Table 3:** Behavioral response times to the incongruent Stroop condition and correlation to glutamate and glutamine concentrations and percent changes during those trials

*Trial*	*Subject group*	*Response time (s)*	*Correlations*							
			*Glu*	*P*	*ΔGlu*	*P*	*Gln*	*P*	*ΔGln*	*P*
Stroop1	HC	0.92±0.16	−0.42	0.077	0.04	0.446	−0.45	0.081	−0.34	0.151
	MDD	0.95±0.12	−0.37	0.08	−0.30	0.126	−0.41	0.063	−0.67	**0.003**
	SZ	1.03±0.08	−0.35	0.099	−0.23	0.207	−0.50	0.033	−0.65	**0.006**
	All	0.97±0.13	−0.32	0.017	−0.15	0.166	−0.40	**0.005**	−0.33	0.019
										
Stroop2	HC	0.84±0.15	−0.39	0.107	0.28	0.178	−0.60	0.026	−0.58	0.031
	MDD	0.89±0.13	−0.30	0.133	−0.28	0.144	−0.20	0.249	−0.45	0.055
	SZ	0.93±0.13	0.38	0.083	0.14	0.309	−0.17	0.287	−0.20	0.244
	All	0.89±0.14	−0.08	0.313	0.04	0.387	−0.28	0.040	−0.28	0.045

Abbreviations: All, the combination of all the participants across groups; HC, healthy control; MDD, major depressive disorder; SZ, schizophrenia.

Glu represents glutamate correlation coefficient (Pearson *r*) with response times. ΔGlu represents the normalized glutamate concentration change. Gln represents glutamine correlation coefficient (Pearson *r*) with response times. ΔGln represents the normalized glutamine concentration change correlation coefficient (Pearson *r*) with response times. *P* represents probability that the correlation is due to chance (alpha=0.05/4 (Bonferroni corrected), one-tailed), values in bold indicate statistical significance.
