# Supplementary material for: Functional magnetic resonance spectroscopy of glutamate in schizophrenia and major depressive disorder: anterior cingulate activity during a color-word Stroop task
Source: NPJ Schizophr. 2015 Sep 16;1:15028–. doi: 10.1038/npjschz.2015.28 (PMC4849454; doi:10.1038/npjschz.2015.28)
Supplement: Supplementary Table 1 [file npjschz201528-s1.doc]

Supplementary Table 1. Pairwise comparisons for adjacent blocks of the ^1^H-fMRS paradigm, normalized to the resting concentration for Glu, Gln, and Glx

| Group | | Resting | | | Stroop1 | | | *p(S1-B)* | Recovery1 | | | *p(R1-S1)* | Stroop2 | | | *p(S2-R1)* | Recovery2 | | | *p(R2-S2)* |
| --- | --- | --- | --- | --- | --- | --- | --- | --- | --- | --- | --- | --- | --- | --- | --- | --- | --- | --- | --- | --- |
| HC | Glu_N | 1.000 | ± | 0.000 | 1.032 | ± | 0.055 | **0.005** | 1.004 | ± | 0.059 | **0.006** | 1.001 | ± | 0.052 | 0.808 | 1.012 | ± | 0.075 | 0.340 |
|  | Gln_N | 1.000 | ± | 0.000 | 1.044 | ± | 0.214 | 0.598 | 1.041 | ± | 0.282 | 0.968 | 0.982 | ± | 0.180 | 0.491 | 1.002 | ± | 0.157 | 0.715 |
|  | Glx_N | 1.000 | ± | 0.000 | 1.033 | ± | 0.060 | **0.013** | 1.012 | ± | 0.062 | 0.027 | 1.003 | ± | 0.043 | 0.602 | 1.015 | ± | 0.075 | 0.363 |
| MDD | Glu_N | 1.000 | ± | 0.000 | 1.016 | ± | 0.049 | 0.082 | 1.003 | ± | 0.059 | 0.101 | 0.963 | ± | 0.055 | 0.005 | 0.969 | ± | 0.035 | 0.634 |
|  | Gln_N | 1.000 | ± | 0.000 | 1.048 | ± | 0.249 | 0.547 | 1.068 | ± | 0.275 | 0.766 | 1.005 | ± | 0.290 | 0.451 | 0.954 | ± | 0.276 | 0.333 |
|  | Glx_N | 1.000 | ± | 0.000 | 1.022 | ± | 0.060 | 0.066 | 1.009 | ± | 0.069 | 0.135 | 0.967 | ± | 0.084 | **0.011** | 0.974 | ± | 0.043 | 0.598 |
| SZ | Glu_N | 1.000 | ± | 0.000 | 1.016 | ± | 0.031 | 0.081 | 1.014 | ± | 0.061 | 0.420 | 0.981 | ± | 0.071 | 0.017 | 0.979 | ± | 0.076 | 0.892 |
|  | Gln_N | 1.000 | ± | 0.000 | 1.264 | ± | 0.386 | **0.001** | 1.048 | ± | 0.381 | **0.002** | 1.071 | ± | 0.313 | 0.778 | 1.087 | ± | 0.326 | 0.757 |
|  | Glx_N | 1.000 | ± | 0.000 | 1.034 | ± | 0.049 | **0.011** | 1.013 | ± | 0.08 | 0.027 | 0.982 | ± | 0.081 | 0.065 | 0.985 | ± | 0.087 | 0.840 |
| All | Glu_N | 1.000 | ± | 0.000 | 1.021 | ± | 0.045 | **0.002** | 1.007 | ± | 0.058 | **0.010** | 0.981 | ± | 0.061 | **0.002** | 0.986 | ± | 0.066 | 0.449 |
|  | Gln_N | 1.000 | ± | 0.000 | 1.124 | ± | 0.309 | 0.014 | 1.053 | ± | 0.311 | 0.088 | 1.021 | ± | 0.267 | 0.490 | 1.016 | ± | 0.266 | 0.865 |
|  | Glx_N | 1.000 | ± | 0.000 | 1.030 | ± | 0.056 | **0.001** | 1.011 | ± | 0.069 | **0.003** | 0.984 | ± | 0.074 | **0.006** | 0.991 | ± | 0.071 | 0.341 |

*p(S1-B)* = Stroop1 vs resting, (alpha=0.05/4 (Bonferroni corrected); one-tailed (Glu, Glx), and two-tailed (Gln)), bolded values indicate statistical significance

*p(R1-S1)* = Recovery1 vs Stroop1, (alpha=0.05/4 (Bonferroni corrected); one-tailed (Glu, Glx), and two-tailed (Gln)), bolded values indicate statistical significance

*p(S2-R1)* = Stroop2 vs Recovery1, (alpha=0.05/4 (Bonferroni corrected); two-tailed (Glu, Gln, Glx)), bolded values indicate statistical significance

*p(R2-S2)* = Recovery2 vs Stroop2, (alpha=0.05/4 (Bonferroni corrected); two-tailed (Glu, Gln, Glx)), bolded values indicate statistical significance

HC = Healthy controls

MDD = Major Depressive Disorder

SZ = Schizophrenia

All = the combination of all participants across groups

Glu_N = The concentration of glutamate normalized to the resting period

Gln_N = The concentration of glutamine normalized to the resting period

Glx_N = The concentration of Glx (glutamate + glutamine) normalized to the resting period
