# Supplementary material for: Functional magnetic resonance spectroscopy of glutamate in schizophrenia and major depressive disorder: anterior cingulate activity during a color-word Stroop task
Source: NPJ Schizophr. 2015 Sep 16;1:15028–. doi: 10.1038/npjschz.2015.28 (PMC4849454; doi:10.1038/npjschz.2015.28)
Supplement: Supplementary Table 2 [file npjschz201528-s2.doc]

Supplementary Table 2. Correlation values between behavioural response times for each Stroop condition and Glu and Gln concentrations during Stroop1

|  |  | Stroop1 | | | | | Stroop2 | | | | |
| --- | --- | --- | --- | --- | --- | --- | --- | --- | --- | --- | --- |
| Group | Metabolite | Congruent | Incongruent | Word Only | Color Only | All | Congruent | Incongruent | Word Only | Color Only | All |
| HC | Glu | -0.394 | -0.419 | -0.152 | -0.614 | -0.471 | -0.551 | -0.454 | -0.085 | -0.558 | -0.494 |
|  | ΔGlu | 0.232 | 0.042 | -0.070 | 0.034 | 0.074 | 0.173 | 0.253 | 0.190 | 0.443 | 0.255 |
|  | Gln | -0.559 | -0.453 | -0.296 | -0.552 | -0.546 | -0.424 | -0.643 | -0.468 | -0.301 | -0.540 |
|  | ΔGln | -0.131 | -0.343 | -0.245 | -0.084 | -0.242 | 0.000 | -0.216 | -0.386 | -0.176 | -0.209 |
| MDD | Glu | -0.009 | -0.368 | -0.390 | -0.276 | -0.304 | 0.076 | -0.270 | -0.181 | 0.013 | -0.123 |
|  | ΔGlu | -0.344 | -0.304 | -0.081 | -0.348 | -0.310 | -0.212 | -0.137 | -0.298 | -0.074 | -0.203 |
|  | Gln | -0.291 | -0.443 | 0.029 | 0.105 | -0.220 | 0.010 | -0.250 | -0.305 | -0.229 | -0.215 |
|  | ΔGln | 0.319 | -0.525 | 0.217 | 0.432 | 0.172 | -0.181 | -0.583 | -0.200 | -0.472 | -0.436 |
| SZ | Glu | 0.091 | -0.352 | 0.192 | 0.129 | 0.028 | 0.115 | 0.334 | 0.002 | 0.454 | 0.302 |
|  | ΔGlu | -0.088 | -0.228 | 0.166 | 0.121 | 0.003 | -0.062 | -0.429 | 0.128 | -0.238 | -0.147 |
|  | Gln | -0.077 | -0.439 | -0.296 | -0.132 | -0.267 | -0.345 | -0.290 | -0.157 | -0.259 | -0.324 |
|  | ΔGln | -0.089 | -0.676 | -0.383 | -0.366 | -0.427 | -0.024 | -0.239 | -0.019 | -0.016 | -0.094 |
| All | Glu | -0.055 | -0.321 | -0.140 | -0.214 | -0.222 | -0.035 | -0.097 | -0.069 | 0.069 | -0.041 |
|  | ΔGlu | -0.092 | -0.149 | -0.017 | -0.131 | -0.115 | -0.021 | -0.092 | 0.033 | -0.113 | -0.063 |
|  | Gln | -0.207 | -0.404 | -0.301 | -0.214 | -0.331 | -0.163 | -0.310 | -0.259 | -0.237 | -0.284 |
|  | ΔGln | -0.003 | -0.330 | -0.150 | -0.024 | -0.154 | 0.017 | -0.213 | -0.068 | -0.010 | -0.092 |

Glu = glutamate correlation coefficient (Pearson r) with response times

ΔGlu = The normalized glutamate concentration change

Gln = glutamine correlation coefficient (Pearson r) with response times

ΔGln = The normalized glutamine concentration change correlation coefficient (Pearson r) with response times

HC= healthy controls

MDD = major depressive disorder

SZ = Schizophrenia

All = the combination of all participants across groups
